# Using shear wave elastography to assess uterine tonicity after vaginal delivery

**DOI:** 10.1038/s41598-021-89756-6

**Published:** 2021-05-17

**Authors:** Joanna Sichitiu, Jean-Yves Meuwly, David Baud, David Desseauve

**Affiliations:** 1 Women – Mother – Child Department, Lausanne University Hospital, Avenue Pierre Decker 2, 1011 Lausanne, Switzerland; 2 Department of Radiology, Lausanne University Hospital, Lausanne, Switzerland

**Keywords:** Medical imaging, Reproductive signs and symptoms

## Abstract

This study aims to evaluate the feasibility and clinical interest of shear wave elastography, by quantitatively estimating the baseline stiffness of the myometrium before and after placental expulsion. We conducted a prospective cohort study of women at term, without known risk factors for postpartum hemorrhage, who gave birth via spontaneous labor in our tertiary center. Myometrium tonicity was evaluated based on measurements of shear wave speed (SWS) in the anterior uterine corpus. All data points were collected by a single operator. Measurements were carried out at three different time points: after fetal delivery (T1), after placental delivery (T2) and 30 min after placental delivery (T3). Our primary objective was to assess the feasibility of this new imaging technique. Ten valid SWS measurements obtained at each of the three different time points were considered as a positive primary outcome. Our secondary objectives were to evaluate the difference in median myometrial shear wave velocity between each time point, as well as to determine the correlation between myometrial shear wave velocity and patients’ characteristics. 38 women were recruited during the study period, of whom 34 met the study criteria. 1017 SWS measurements were obtained. The median time to perform measurements was 16 s for one value, and 2 min 56 s for ten. For 11 women (32%) it was not possible to achieve ten SWS at T1 as placental expulsion immediately followed the birth of the newborn. One patient experienced placental retention and only measurements at T1 were performed. For all other patients, we were successfully able to obtain all measures as intended. There was no difference in the mean shear wave speed between the three time points. After adjustments for confounders, we observed a significant correlation for total blood loss (correlation coefficient = − 0.26, *p* < 0.001, units of oxytocin (correlation coefficient = − 0.34, *p* = 0.03), and newborn weight (correlation coefficient = − 0.08, *p* = 0.001). It is feasible to assess uterine tonicity by shear wave imaging, after placental expulsion. We did not observe a variance in uterine tonicity between the three time points. Women who had higher blood loss, received more units of oxytocin and/or those with newborns of a higher weight exhibited lower shear wave speed measures.

## Introduction

Postpartum hemorrhage (PPH) is critically dependent on myometrial contraction and relaxation during and following the third stage of labor. In the absence of this physiological process, uterine atony leads to significant maternal morbidity and mortality^1^. Uterine atony accounts for more than two thirds of all cases of PPH. However, compared to other common causes, such as maternal perineal laceration, coagulopathy and retained placenta, controlling uterine atony proves more challenging^2–5^. Even though multiple risk factors of uterine atony have been described throughout the literature (past history of PPH, grand multiparity, multiple pregnancy, hydramnios, fetal macrosomia, use of oxytocin for labor induction or augmentation, labor induction, prolonged labor, protracted second stage of labor, chorioamnionitis, obesity, previous uterine scar)^3–8^, it often occurs among patients with no risk factors^5^. Indeed, predictive probability of uterine atony remains low^6,9^. Identification of at-risk women would allow us to anticipate PPH, apply appropriate preventative and therapeutic measures, and lead to a reduction in adverse maternal outcomes^10^. Instead of monitoring uterine contraction, which exhibits variance between women^11–13^, we hypothesize that baseline uterine muscle tone could predictive of uterine atony. Evaluation of uterine tonicity by manual palpation has been shown to be subjective and inaccurate^14,15^, highlighting the requirement for new, accurate and easy-to-use technologies in this field.

In the last few years, studies on the application of elastography in obstetrics have focused on the prediction of preterm delivery^16,17^, success of labor induction^16^, elasticity change of the myometrium during labor^18^ and placenta pathologies^19,20^. Elastography, both the quasi-static (strain) and dynamic methods, allows a biomechanical definition tissue stiffness by evaluating the correlation between stress and strain i.e. the force applied to and the resultant deformation of the tissue^21^. Quasi-static elastography involves manipulation of the transducer to compress the tissue, with an elastogram illustrating the degree of deformation between this and the neighbouring unstressed tissue. The subjectivity of this method is a major disadvantage, with human factors playing a large role in both data acquisition and analysis^21^. The methods necessary for dynamic elastography, by comparison, are more objective and easily reproducible, as they are independent of operator variance^21^. A shear wave is produced by applying an acoustic force to the tissues, and its speed is determined by ultra-speed ultrasound scan. Either acoustic radiation force (i.e. point shear wave elastography and 2-D shear wave elastography) or external vibrators may be used to manufacture the shear wave^21^. Shear wave speed (SWS) is proportionally related to tissue stiffness; the stiffer the tissue, the faster the wave’s propagation speed^21^. Tissue elasticity evaluation can be expressed as either shear wave speed (m/s) or Young’s Modulus (kPa). The Young’s Modulus (E) can be directly calculated from the shear wave velocity (V_s_ in m/s) by the equation E = 3*p*V_s_^2^, *p* being the density (~ 1000 kg m^−3^)^18^. However, this calculation is only valid in an isotropic medium, and the myometrium may be considered anisotropic^18^. Therefore, in this study tissue elasticity will be expressed in terms of shear wave speed.

Despite shear wave elastography being a widely used imaging technique in a range of medical fields, some limitations exist. Indeed, to obtain an accurate measurement of shear wave speed, there is a need for satisfactory displacement of tissue by the shear waves as well as an adequate signal‐to‐noise ratio^22^. These can be affected by a number of factors such as: limited penetration, reverberations, tissue architecture and anisotropy, motion artefacts, and pulsatile artefacts due to large blood vessels^23,24^. In this context, a prospective study is warranted to assess the technical feasibility of myometrium shear wave velocity measurement and its correlation to blood loss in the postpartum period before any clinical applications of this technique.

The primary objective of this study was to evaluate the feasibility and clinical interest of shear wave elastography, by quantitatively estimating the baseline stiffness of the myometrium during the third stage of labor and after placental expulsion.

## Material and methods

### Study population

This is a prospective feasibility study conducted in the Maternity unit of the Lausanne University Hospital between June and November 2019. Women without known risk factors for PPH, above 37 weeks of gestation and giving birth after a spontaneous labor in our center were invited to participate. As limited data exists concerning evaluation of myometrial stiffness by elastography during the third stage of labor and after placental expulsion, patients at higher risk of uterine atony were excluded. Therefore, the exclusion criteria were as follows: multiparity, protracted second stage of labor (more than 3 h from full dilatation to delivery), administration of more than 3 units of oxytocin during the second stage of labor, more than 30 min of active pushing, BMI > 35 kg/m^2^, women above 35 or under 18 years old, induction of labor, instrumental deliveries, antepartum hemorrhage in the current pregnancy, polyhydramnios, diabetes, pre-eclampsia, high blood pressure, clinical chorioamnionitis, placental abnormality (low-lying or abruption), fetal macrosomia, uterine fibroids, uterine anomalies, previous uterine scar, multiple pregnancy, bleeding disorders, and use of anticoagulant medications. In our unit, as per protocol, women stay at full dilatation for up to 3 h before active pushing. Active management of the third stage of labor was performed for every patient, by routinely administrating a prophylactic 5 UI intravenous oxytocin bolus after delivery of the anterior shoulder. The patient was monitored for 2 h after placental delivery in the labor suite, as usual.

All participants provided written informed consent before the shear wave measurements were performed. This study was approved by the local IRB (Ethical Commission of the Canton of Vaud, Switzerland, 2018-02267). All methods/experiments were carried out in accordance with relevant guidelines and regulations (Declaration of Helsinki).

### Measurements

**Figure 1:**
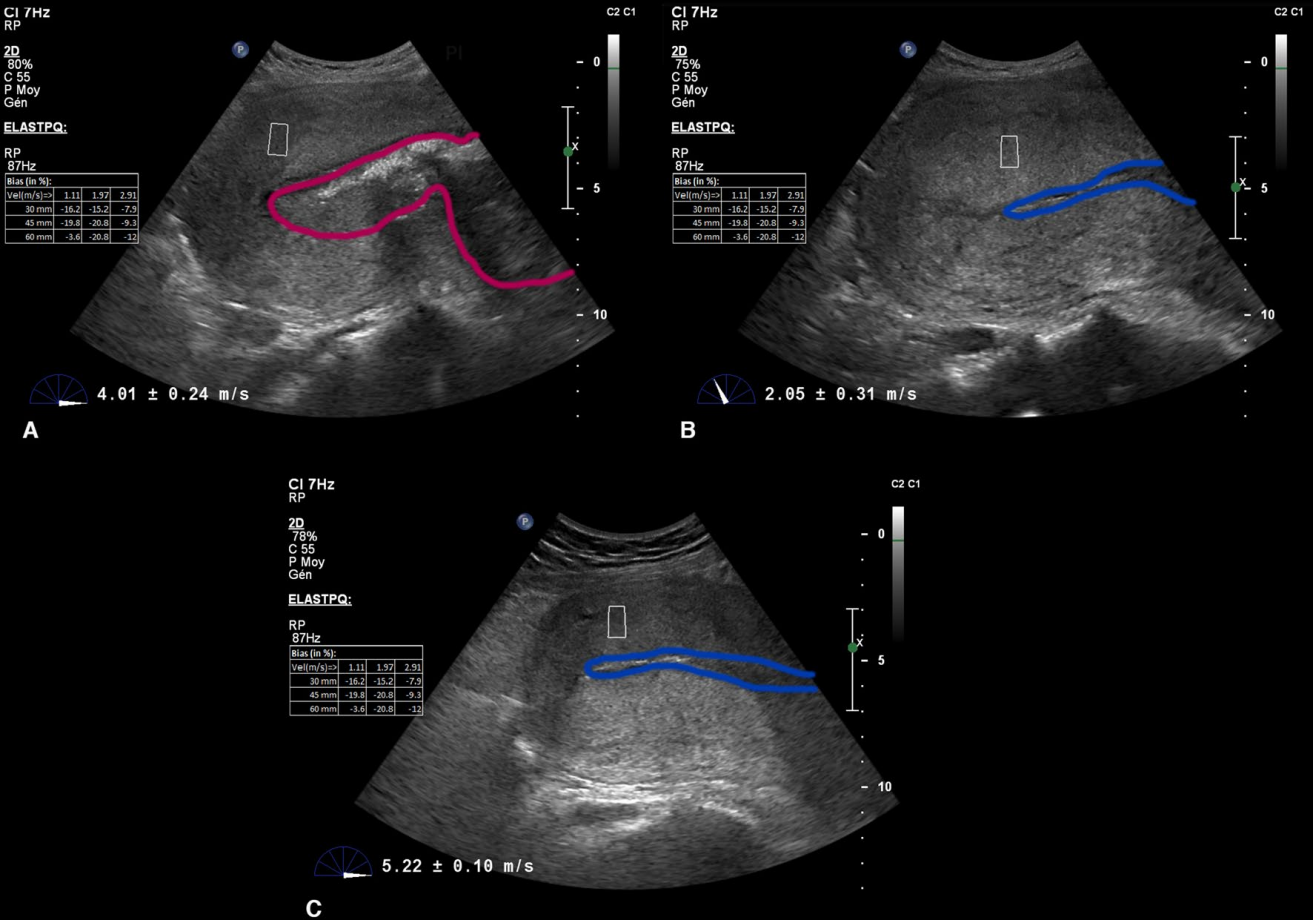
Ultrasound shear wave elastography images. Shear wave measurement at the different time points. (**A**) Before placental expulsion. (**B**) Immediately after placental expulsion. (**C**) 30 min after placental expulsion. Pink outline illustrates the placenta. Blue outline illustrates the uterine cavity.

The point shear wave measurements were performed using the ElastPQ module on a Philips iU22 ultrasound system with a C5-1 curvilinear transducer (1 to 5 MHz). The measurements were carried out by the same operator at three different time points: after fetal delivery (T1), after placental delivery (T2) and 30 min after placental delivery (T3) (Fig. 1). The operator was able to select the region of interest (ROI) over a B-Mode US image. The ElastPQ module permits only a predefined size of ROI (15 × 5 mm). The myometrium was sonographically identified as a homogeneous echogenic layer between the serosa and the decidua^25^. A mid-sagittal view was performed with continuous visualization of the uterus. To assure consistency in obtaining a mid-sagittal plane of the uterus when the placenta was in situ, the maternal abdominal aorta was used as a reference point, as previously described^25^. After placental expulsion, a continuous and linear endometrial stripe was the preferred reference point to ensure a midsagittal view (Supplementary Figure S1). SWS was determined at a single site, at the anterior uterine corpus. Each measurement was taken halfway between the serosa of the uterus and the endometrial cavity. At least 10 valid measurements were performed, maintaining the probe in place for each assessment, and quantified in meters per second. If measurement reliability was low, “0.00” was displayed on the screen, meaning tissue could not be detected. If 10 valid measurements are not obtained after 15 attempts, the assessment was deemed unsuccessful. The maximum depth at which the ElastPQ system can calculate shear wave speed is 8 cm^26^. Distance between the skin and the uterine point of focus was therefore sonographically measured.

### Outcomes

Our primary objective was to assess the feasibility of shear wave elastography of the uterus in the third stage of labor and immediate postpartum. Ten valid SWS measurements obtained at each of the three different time points (T1, T2, T3) were considered as a positive primary outcome. Secondary outcomes included: the difference in median myometrial shear wave velocity between each time point and the correlation between myometrial shear wave velocity and other variables (total blood loss, maternal age, BMI, body weight, depth of measurement, gravidity, gestational age, labor duration, duration of second stage and third stage, duration of active pushing, units of oxytocin given throughout labor and newborn weight).

Relevant demographic, obstetrical and neonatal characteristics were collected from the patients’ medical charts. Total blood loss was quantified by collector bags placed under the mother’s pelvis after fetal delivery, and then with sanitary pads once the placenta was delivered and perineal tear suturing was completed. Measurement of blood loss by sanitary pads was performed by weighing the pads on a digital scale.

### Statistical analysis

The power calculation was based on the study performed by Tanaka et al., the only report to date investigating uterine shear wave velocity in the postpartum period^27^. We calculated that 38 patients would provide the study with a power of 90%, assuming that the mean uterine shear wave speed after placental expulsion would be 33% higher than the before the expulsion, with a standard deviation of 0.85 and a two-sided, type 1 error rate of 0.05.

Continuous data was reported as mean ± SD or median (interquantile range). A Shapiro Wilk test was used to assess the normality of the distribution of variables. As distribution was not normal, difference in myometrial shear wave velocity between each time point (after fetal delivery, after placental delivery and 30 min after placental delivery) was tested for statistical significance using the Kruskal–Wallis test. Variance was used to assess the variability in our SWS measurements.

The coefficient of determination R^2^ between shear wave velocity and total blood loss was assessed by simple linear regression. The correlation coefficient between myometrial shear wave velocity and other variables (total blood loss, maternal age, BMI, body weight, depth of measurement, gravidity, gestational age, labor duration, duration of second stage and third stage, duration of active pushing, units of oxytocin given throughout labor and newborn weight) was assessed by using a generalized linear mixed model (GLMM) with a univariate and multivariate analysis. Variables included in the multivariate analysis were selected according to their clinical relevance and had a *p* value of less than 0.20 in the univariate analysis.

A two tailed *p* value of less than 0.05 was considered to indicate statistical significance. All analyses were performed using STATA software (version 14IC; Stata corporation, College Station, TX).

## Results

During the study period, a total of 38 women were recruited to participate. However, only 34 fulfilled the inclusion criteria as 4 women had an instrumented vaginal delivery. Demographic, obstetrical and neonatal characteristics of participants are illustrated Table 1. The median age was 30 years, gestational age 40 weeks, and BMI was 26.8 kg/m^2^. 28 women (82.4%) were given an intravenous infusion of oxytocin during the second stage of labor, with a median 0.7 units. The median total blood loss was 260 ml. Two women (5.8%) presented with post-partum hemorrhage, one during the following 2 h surveillance period in the labor ward, and the other in the context of placental retention.

**Table 1:** Demographic, obstetrical and neonatal characteristics.

Demographic and baseline characteristics (n = 34)	
Maternal age (years)	30 [6]
Gestational age (weeks)	40 [2]
Gestity	1.0 [2]
BMI (kg/m^2^)	26.8 [3.2]
Depth of measurement (cm)	4.8 [0.8]
Units of oxytocin (UI)	0.7 [0.9]
Blood loss (ml)	260 [180]
Newborn weight (g)	3395 [440]
Labor duration (min)	300 [204]
Duration passive 2nd stage (min)	150 [60]
Duration active 2nd stage (min)	18 [18]
Duration 3rd stage (min)	12 [12]

Data are presented as median values [interquartile range].

A total of 1017 SWS measurements were obtained. For 11 women (32%), it was not possible to measure 10 shear wave velocities at T1 as placental expulsion immediately followed the birth of the newborn, allowing insufficient time to perform the ultrasound examination. However for these 11 patients, measurements at T2 and T3 were successful. As mentioned above, one patient experienced placental retention complicated by postpartum hemorrhage and only the first SWS measurement (before placental expulsion) was performed. For all other patients, we were successfully able to obtain all required data points.

**Figure 2:**
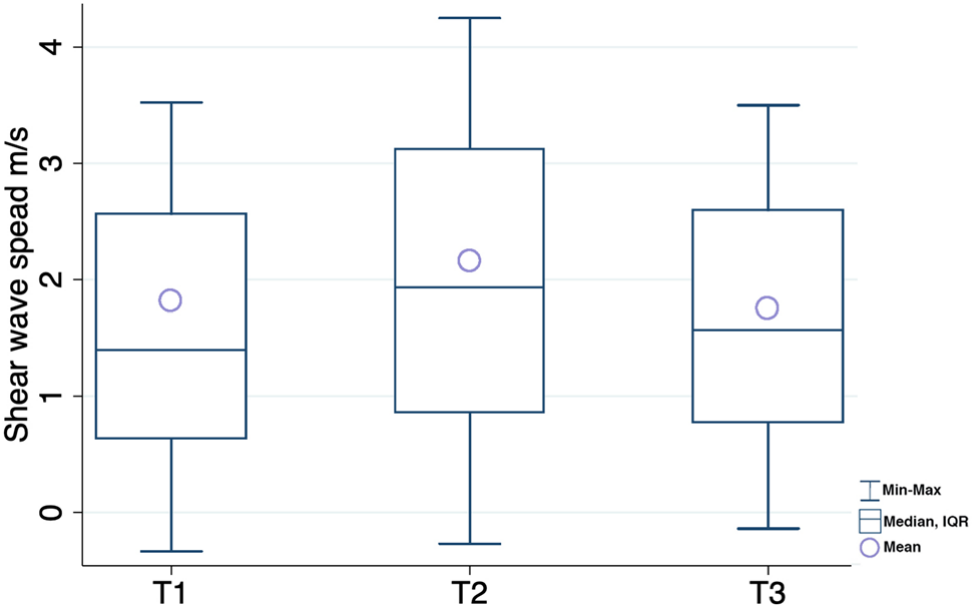
Box‐and‐whisker plot showing shear wave speed mean and median at the three different time points. T1: measures after fetal delivery, T2: measures after placental delivery, T3: measures 30 min after placental delivery.

A large variation in shear wave velocities was observed for all patients and time points (Fig. 2). The median acquisition time to obtain 10 valid measurements was 3 min 01 s for T1, 2 min 57 s for T2, and 2 min 46 s for T3. The overall mean SWS was 1.91 ± 1.36 m/s with a variance of 1.86. Mean SWS was 1.8 ± 1.5 m/s, 2.2 ± 1.4 m/s, and 1.7 ± 1.2 m/s for T1, T2 and T3 respectively. The variance at each time was similar. There was no significant difference in the shear wave speed measured at the uterine anterior corpus between the three different time points (*p* = 0.49).

**Figure 3:**
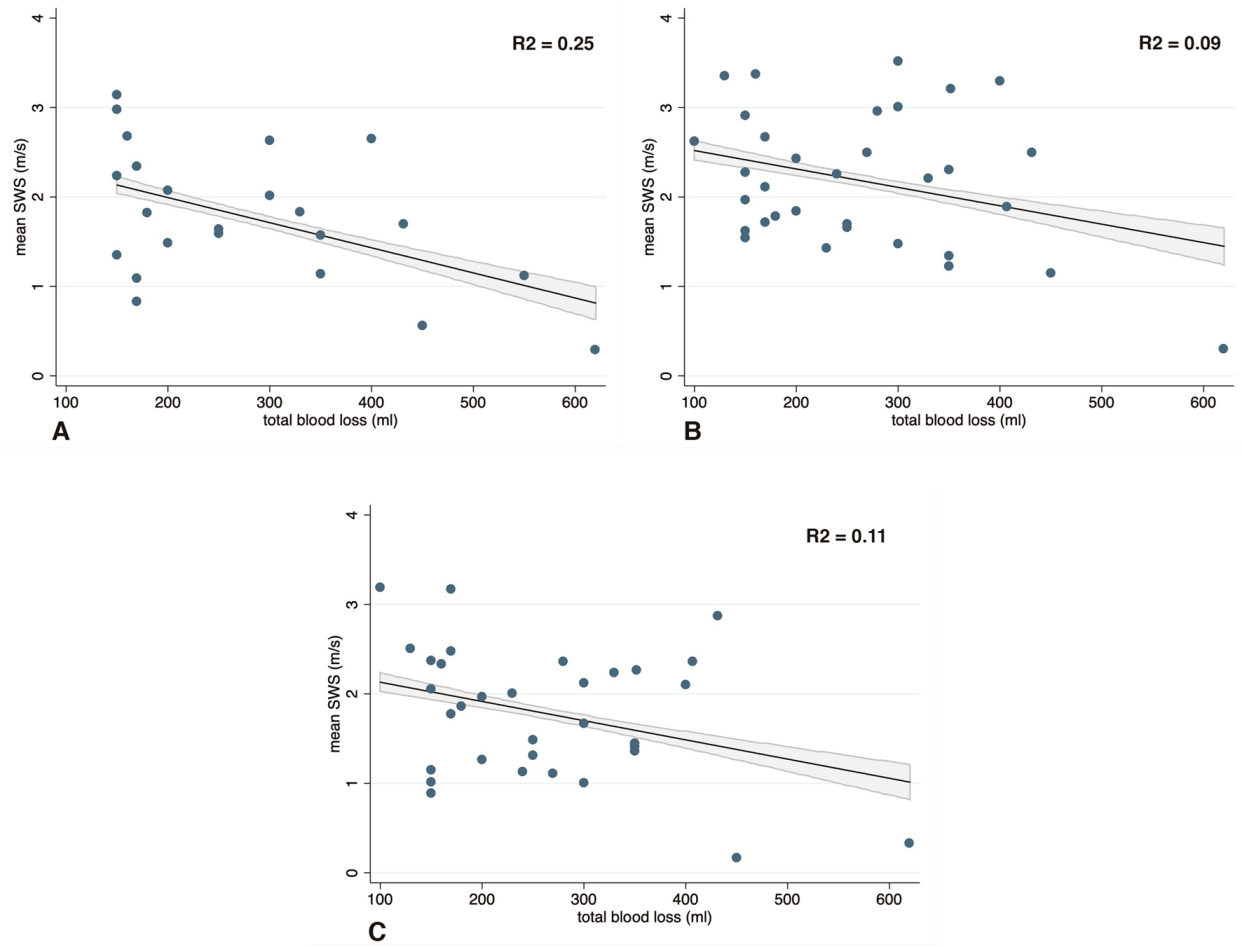
Mean SWS and total blood loss. Relationship between mean SWS and total blood loss between at the three different time points. The figure illustrates the ‘‘FitPlot’’ consisting of scatter plots of the data overlaid with the regression line, and 95% confidence interval. Correlation coefficients are reported (R^2^). (**A**) Before placental expulsion. (**B**) Immediately after placental expulsion. (**C**) 30 min after placental expulsion.

We found a significant relationship, by simple linear regression, between total blood loss and mean SWS before placental expulsion (*p* < 0.001), but with a low R^2^ value (0.25) suggestive of variability, as illustrated in Fig. 3a. Similar observations were made for mean SWS, both after placental expulsion (*p* < 0.001, R^2^ = 0.09, Fig. 3b) and at 30 min after placental delivery (*p* < 0.001, R^2^ = 0.11, Fig. 3c). In the generalized linear mixed model, the correlation between mean SWS and total blood loss remained significant in univariate (*r* = − 0.27, *p* < 0.001) and multivariate analysis (*r* = − 0.26, *p* < 0.001). Concerning the other parameters, the univariate analysis illustrated a correlation between mean SWS and BMI (*r* = − 0.08, *p* < 0.001), depth of measurement (*r* = − 0.50, *p* < 0.001), units of oxytocin (*r* = − 0.20, *p* = 0.008), and newborn weight (*r* = − 0.05, *p* = 0.005). After adjustments for confounders, we observed a correlation only for units of oxytocin (*r* = − 0.34, *p* = 0.03), and newborn weight (*r* = − 0.08, *p* = 0.001) as shown in Table 2.

**Table 2:** Correlation coefficients with mean SWS.

	Univariate analysis		Multivariate analysis	
	Correlation coefficient with mean shear wave speed (95% CI)	*p*	Correlation coefficient with mean shear wave speed (95% CI)^a^	*p*
Maternal age	− 0.01 [− 0.06; 0.04]	0.67		
Gestational age	0.002 [− 0.22; 0.23]	0.98		
BMI	− 0.08 [− 0.12; 0.03]	< 0.001	− 0.05 [− 0.13; 0.02]	0.10
Depth of measurement	− 0.50 [− 0.67; − 0.33]	< 0.001	− 0.18 [− 0.50; 0.13]	0.27
Labor duration	− 0.04 [− 0.09; 0.002]	0.05	0.04 [− 0.04; 0.11]	0.55
Duration passive 2nd stage	− 0.98 [− 0.23; 0.03]	0.13	0.02 [− 0.02; 0.05]	0.64
Duration active 2nd stage	− 0.18 [− 0.79; 0.42]	0.55		
Duration 3rd stage	− 0.56 [− 1.18; 0.06]	0.07	− 0.40 [− 1.32; 0.53]	0.67
Units of oxytocin	− 0.20 [− 0.35; − 0.05]	0.008	− 0.34 [− 0.62; − 0.05]	0.03
Total blood loss	− 0.27 [− 0.35; − 0.19]	< 0.001	− 0.26 [− 0.41; − 0.11]	< 0.001
Newborn weight	− 0.05 [− 0.08;− 0.01]	0.005	− 0.08 [− 0.12; − 0.03]	0.001

*BMI* body mass index, *CI* confidence interval.

^a^Adjusted for body mass index, depth of measurement, labor duration, duration passive 2nd stage, duration 3rd stage, units of oxytocin, total blood and newborn weight.

## Discussion

In this prospective study, we illustrated that measurement of SWS at the uterine anterior corpus after placental expulsion is feasible. Furthermore, the association analysis showed correlations between SWS and units of oxytocin administered, newborn’s weight, as well as total blood loss. No significant difference was found in the mean SWS between the three time points.

Physiological changes of the uterus in the postpartum period are still not yet fully understood^28^. The description in the literature of normal uterine involution focuses on either maternal palpation or ultrasound measures of the uterus^28,29^. By contrast, we described the basal myometrial tone quantitatively, and its evolution throughout the third stage of labor as well as the immediate postpartum period. It was not possible to perform measurements between fetal delivery and placental expulsion in a large part of our population (32%), given an insufficient time interval of less than 2 min. Thus, it was deemed not feasible to evaluate the SWS before placental expulsion. Otherwise, we were able to obtain ten measurements immediately following placental expulsion and 30 min after placental delivery.

This study is the first to report known risk factors of uterine atony (units of oxytocin received and newborn weight), with reference to an objective quantification of uterine stiffness. We specifically selected a patient cohort presenting with a low risk for uterine atony, as we wanted to obtain data on uterine elasticity in a physiological third stage of labor and immediately post-partum. Therefore, we could not investigate the association between myometrial elasticity and other classical risk factors, such as labor dystocia, increased maternal age, multiple pregnancy, induction of labor and hydramnios^3^.

We need to be cautious with the interpretation of our results for the following reasons. Only one previous study has investigated uterine stiffness by elastography during the third stage of labor and in the postpartum period. Tanaka et al.^27^, examined shear wave velocity in the uterine corpus before, immediately after, and 1 and 2 h after placental delivery in 11 patients. In contrast to our results, the authors illustrated that the stiffness of the uterine corpus changed significantly over time, and was significantly higher immediately following, and 1 and 2 h after placental delivery, as compared with before placental delivery. These contraindicatory results may be due to the presence of variability in shear wave speed measurement between our study and that of Tanaka et al.^27^ The authors did not report such variability in their measurements, however only 3 valid measures were performed per patient, in contrast to the 10 measures performed in our study. Furthermore, similar variability in SWS was found in studies regarding the myometrial elasticity during labor^18^, and cervical elasticity^30^. It has been previously illustrated that shear wave propagation is highly sensitive to tissue anisotropy, which might explain this variability^31,32^. In contrast to an isotropic medium such as that found in the liver, where the shear waves propagate at the same speed in any direction, this is not the case in anisotropic tissue. Indeed, in anisotropic media such as the myocardium, brain, muscle and uterus, the fibers’ structure induce different shear wave speeds along the direction of propagation^31,32^. SWS are higher when travelling parallel rather than perpendicular to the fibers^31^. Magnetic resonance diffusion tensor imaging illustrated that anisotropy is present in most parts of the uterus^33,34^. A preliminary study on one pregnant patient also showed the presence of anisotropy by 2-D shear wave elastography, demonstrating variation of uterine elasticity as a function of the angle of rotation of the probe^35^. Lee et al. were able to identify the fiber orientation in the myocardium using shear wave imaging by rotating the transducer at different angles around a symmetrical axis. In this study, anisotropy was not explored, as we did not alter the angle of the probe during measurements. Further studies are needed to explore this question, in order to standardize elastographic measurement of the uterus.

Another explanation for this variation in our SWS measurements is the possible cyclic variation of uterine contraction during the immediate post-partum period, which was neither explored in our study nor by Tanaka et al^27^. It has been shown that during this period, uterine contractions were variable in intensity and frequency^13,36^. The uterus was relaxed between contractions. Some measurements may have been taken during contractions, while others not. A simple investigation would be to monitor the uterine activity by tocodynamometer or intrauterine pressure catheters in parallel to SWS measurement, as a correlation between elastographic and tocometric values have been demonstrated^18^.

We should bear in mind that despite shear wave imaging being an interesting technique, as a safe, quick and non-invasive tool to appraise tissue elasticity, it has shown some drawbacks in studies of other anisotropic organs. The kidney represents an apt example. Multiple articles have been published regarding the investigation of the normal and abnormal kidney by this technique, which resulted in conflicting results regarding its clinical usefulness and reproducibility^22,31^. Currently, some authors question if any of the literature on renal shear wave elastography is accurate^22^. Unique shear wave generation pulsing technology with specific processing algorithms adapted to the kidney are being developed, and may be able to overcome the issue of SWS variability^22^. Similar processes may be required with regard to the investigation of uterine elasticity.

Furthermore, previous phantom studies and on healthy volunteers reported that the depth of measurement had an influence on the shear wave speed^37^. In our study, the median depth of measurement was 4.8 cm (IQR 0.8). We did not find a significant correlation between depth of measurement and SWS in the multivariate analysis. This is concordant with other reports, as both consistency and success rates of stiffness measurements were greater at 3–5 cm from the probe, compared to relatively shallower (< 3 cm) or deeper (> 5 cm) positions^37^. Acquisition of elastographic imaging at deeper locations could be difficult due to decreased accuracy and reliability, which could limit the usage of this technique with obese parturients.

## Conclusion

It is feasible to assess uterine stiffness by shear wave imaging, after placental expulsion. However, investigation of the elasticity of the uterus is still in its pioneer phase, with the presence of high variability in measurements. Further studies are required to enhanced the accuracy of this technique before the implementation in clinical settings, notably by developing a specific shear wave elastography processing algorithm adapted to the viscoelastic proprieties of the gravid uterus.

## Supplementary Information

Supplementary Information.
