# Supplementary material for: Using shear wave elastography to assess uterine tonicity after vaginal delivery
Source: Sci Rep. 2021 May 17;11:10420. doi: 10.1038/s41598-021-89756-6 (PMC8129155; doi:10.1038/s41598-021-89756-6)
Supplement: Supplementary file 1 — Supplementary Information. [file 41598_2021_89756_MOESM1_ESM.pdf]

SUPPLEMENTARY INFORMATION

Title: Using shear wave elastography to assess uterine tonicity after vaginal delivery

Joanna SICHITIU MD,[1] Jean-Yves MEUWLY MD,[2] David BAUD MD-PhD,[1]

David DESSEAUVE MPH MD PhD[1]

Figure S1 Midsagittal view of the uterus

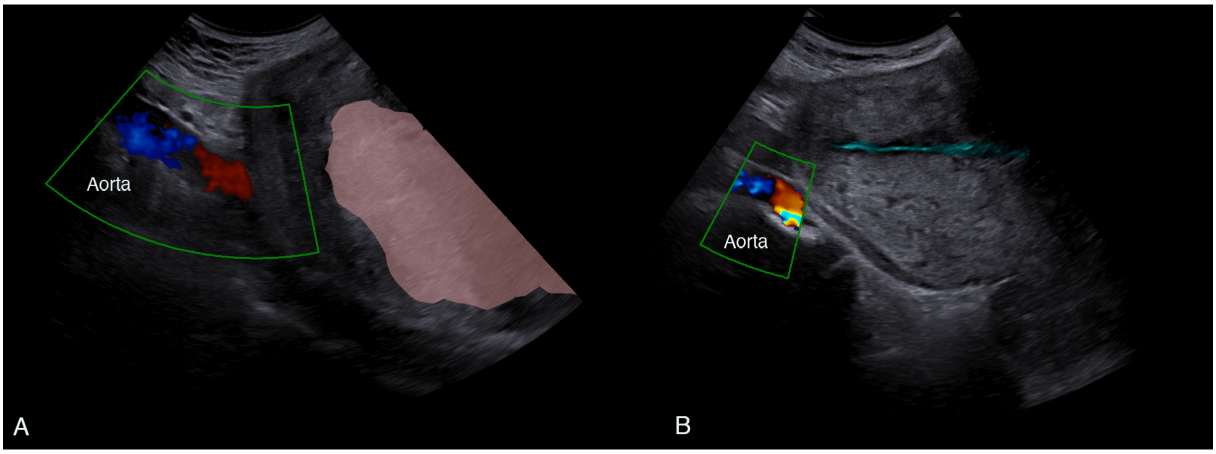

A. Before placental expulsion. B After placental expulsion. Pink outline illustrates the placenta. Blue outline illustrates the uterine cavity.
